# Sex and species specific isotopic niche specialisation increases with trophic complexity: evidence from an ephemeral pond ecosystem

**DOI:** 10.1038/srep43229

**Published:** 2017-02-24

**Authors:** Tatenda Dalu, Ryan J. Wasserman, Tim J. F. Vink, Olaf L. F. Weyl

**Affiliations:** 1 Zoology and Entomology, Rhodes University, Grahamstown, Eastern Cape, South Africa; 2 South African Institute for Aquatic Biodiversity, Grahamstown, Eastern Cape, South Africa; 3 School of Science, Monash University Malaysia, Jalan Lagoon Selatan, 47500 Bandar Sunway, Selangor Darul Ehsan, Malaysia; 4 Department of Botany, Coastal and Marine Research Unit, Nelson Mandela Metropolitan University, P O Box 77000, Port Elizabeth 6031, South Africa

## Abstract

It is generally accepted that organisms that naturally exploit an ecosystem facilitate coexistence, at least partially, through resource partitioning. Resource availability is, however, highly variable in space and time and as such the extent of resource partitioning must be somewhat dependent on availability. Here we test aspects of resource partitioning at the inter- and intra-specific level, in relation to resource availability in an atypical aquatic environment using an isotope approach. Using closely related key organisms from an ephemeral pond, we test for differences in isotopic signatures between two species of copepod and between sexes within each species, in relation to heterogeneity of basal food resources over the course of the ponds hydroperiod. We show that basal food resource heterogeneity increases over time initially, and then decreases towards the end of the hydroperiod, reflective of the expected evolution of trophic complexity for these systems. Resource partitioning also varied between species and sexes, over the hydroperiod with intra- and inter-specific specialisation relating to resource availability. Intra-specific specialisation was particularly evident in the omnivorous copepod species. Our findings imply that trophic specialisation at both the intra- and inter-specific level is partly driven by basal food resource availability.

Empirical studies of resource competition routinely show that niche differences promote coexistence in nature^1^,^2^,^3^. As such, it is now well recognized that species coexistence is, at least partially, facilitated by the partitioning of resources such as food and space^1^. However, in addition to inter-specific differences, many species also show marked trait differences between the sexes, such as body size, morphological traits, feeding or micro–habitat utilisation^4^,^5^,^6^. As a result, male and female organisms within a species can also occupy subtly different “niches”. Given that these demographic groups within a population often have the potential to play different functional roles within an environment, trait differences among sexes can also represent important ecological considerations, particularly when sex ratios are non–Fisherian. Indeed, across a range of ecosystems globally, sex ratios have been observed to vary considerably with sex–skewed ratios commonly encountered^5^,^7^,^8^. With regard to feeding, over and above taxa or sex specific differences, consumer foraging is dynamic – changing in relation to various factors such as individual physiological state or basal food resource availability^9^. Considering such factors is crucial for our understanding of ecological processes in natural and artificial systems.

Ephemeral aquatic environments and the effects of hydroperiod have been well studied globally^10^,^11^,^12^. It is now well recognized that over the course of an ephemeral ponds hydroperiod there is generally a change in trophic complexity, linked to changes in diversity associated with the internal process of dormant eggs hatching and invasions of organisms from external environments^13^,^14^,^15^. This trophic complexity generally increases initially after a pond fills before reaching a crescendo and subsequent decrease as a pond dries out^15^. However, while the invertebrate communities differ substantially over the course of a pond’s hydroperiod, there are those key organisms that persist throughout. These organisms are therefore able to cope not only with changes in the size, depth and water chemistry^16^, but also with associated changes in basal food resources. The present study focusses on two such species and addresses the question of whether these organisms vary food resource exploitation as a result of the rapidly changing environment in which they reside.

In the present study, we assessed both inter– and intra–specific differences between key copepod species in an atypical aquatic environment using an isotope approach. More specifically, we were interested in whether two closely related species assume similar trophic roles in ephemeral aquatic ecosystems and whether within each species, adult males and females were functionally similar over time. Ephemeral pond environments are unique aquatic habitats in that they have discrete wet and dry phases. As a result, community succession is rapid and a major consideration in these environments in that aquatic communities essentially evolve from a state of complete absenteeism, through various levels of complexity and back again, sometimes within a very short period^15^. These environments are, therefore, ideal for assessing aspects of trophic ecology, particularly with regard to temporal dynamics given that aquatic trophic complexity functionally evolves from and to a point of truancy.

In austral regions, the pioneer ephemeral pond communities are dominated by crustaceans such as copepods and daphniids which hatch from dormant eggs^17^,^18^. In the arid regions of South Africa, many of the hatching copepods are representatives of the subfamily Paradiaptominae^19^, a group of large bodied copepods, some of which assume a predatory role in temporary water bodies, while others are thought to be more omnivorous^18^,^20^. Two members of this subfamily, *Lovenula raynerae* Suárez–Morales, Wasserman, Dalu, 2015 and *Paradiaptomus lamellatus* Sars, 1895 are key species in ephemeral ponds of the region. Although these organisms are considered pioneers in that they are among the first to hatch from dormant eggs, they often also persist to near the end of a pond’s hydroperiod^18^,^20^ making them important contributors to trophic dynamics in these environments. While both copepods are considered important consumers in these systems, information on the trophic ecology of these species is largely lacking^18^,^20^. As with other diaptomid copepod species, both *L. raynerae* and *P. lamellatus* are sexually dimorphic, with the adult males of both species being, among other things, slightly smaller than the adult females^21^. As such, we expect that these differences may have trophic niche implications, as has been observed for in other groups^5^,^22^,^23^,^24^.

Given the rapid environmental and biological changes over the course of an ephemeral pond’s typical hydroperiod, and its known implications for overall trophic complexity^15^, there is likely variability in basal resource availability for organisms that persist throughout. We postulate that the key copepod species, *Lovenula raynerae* and *P. lamellatus* will exhibit predictable dietary shifts over the course of a ponds hydroperiod. More specifically, we hypothesise that (1.) at the early stages of a ponds hydroperiod, males and females of both species would have large dietary niche overlaps given the low overall trophic complexity within these systems at this stage, (2.) with an increase in pond age, an increase in intra- and interspecific feeding specialisation will be observed and (3.) at the end of a ponds hydroperiod, known reductions in trophic complexity would once more result in intra- and inter-specific homogenisation of trophic niches for male and female *L. raynerae* and *P. lamellatus*. These hypotheses were tested on a natural plankton community using an isotope approach, whereby the isotopic mixing space of the planktonic food web were determined at four discrete periods over the course of an ephemeral pond’s hydroperiod, in relation to the trophic niche space of male and female *L. raynerae* and *P. lamellatus*.

## Results

### Potential food sources and consumer stable isotope variation

The potential basal food resources and consumers stable isotope values (both δ^13^C and δ^15^N) differed significantly (*p* < 0.05) over the course of the hydroperiod (see Figure S1). Terrestrial organic matter i.e. the grass, *Sporobolus* and detritus were δ^15^N depleted and δ^13^C enriched at survey 1. Over the course of the hydroperiod, the grass became δ^15^N enriched. Particulate organic matter (POM) showed δ^15^N variation whereas sediment showed variation of both δ^15^N and δ^13^C over the four survey periods (Figure S1). In relation to the changes in plant isotopic composition, invertebrates e.g. *Daphnia* spp., Copepoda (*Mesocyclops* sp. and nauplii), *Cyzicus* spp., *Cypricercus* spp. and *Lynceus* spp. showed strong variation in both the δ^15^N and δ^13^C values. With the δ^15^N and δ^13^C values showing initial depleted before being enriched (Figure S1).

**Figure 1:**
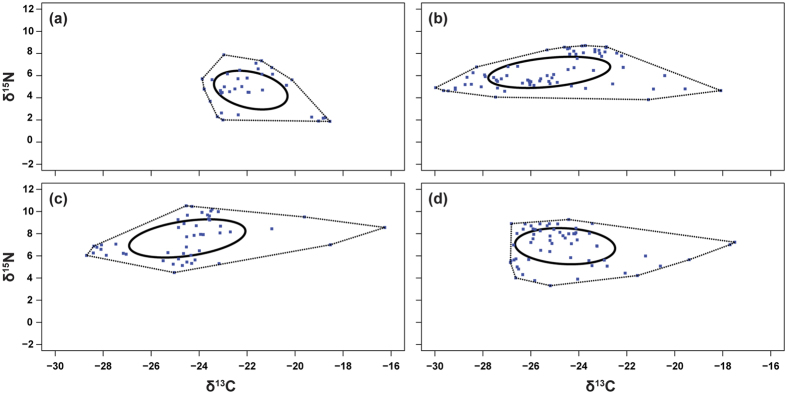
Trophic diversity over the course of the hydroperiod: (**a**) survey 1, (**b**) survey 2, (**c**) survey 3 and (**d**) survey 4, depicted by the convex hull areas (dotted lines) and standard Bayesian ellipses (SEA; solid lines).

The overall mean dNr (δ^15^N range), dCr (δ^13^C range), mean distance to the centroid (CD), mean nearest neighbour distance (MNND), standard deviation of nearest neighbour distance (SDNND and standard ellipse area (SEAc) of the plankton food webs as a whole increased from survey 1 to 3 before decreasing at survey 4 (Fig. 1; Table S1), highlighting changes in trophic complexity and diversity over the course of the hydroperiod. The survey 1 consumer foodweb had a small TA area, with a stunted diamond shaped structure (Fig. 1a). The survey 2 consumer foodweb was, however, more triangular shaped, being shorter and wider along δ^15^N and δ^13^C axis, respectively (Fig. 1b). The consumer foodweb for survey 3 exhibited a trapezoid (i.e. kite-) shape, being wider along the δ^13^C axes (Fig. 1c), while the survey 4 consumer foodweb once again resembled a triangular shape, being shorter along both the δ^15^N and δ^13^C axes when compared to survey 3 (Fig. 1d). The SEAc captured the differences in isotopic trophic niche space over the hydroperiod, with the δ^15^N and δ^13^C axes being smaller in survey 1, whereas a larger area along the δ^13^C axis was observed in surveys 2 and 3. A larger area towards the δ^15^N axis was, however, observed in survey 4 (Fig. 1; Table S1).

**Figure 2:**
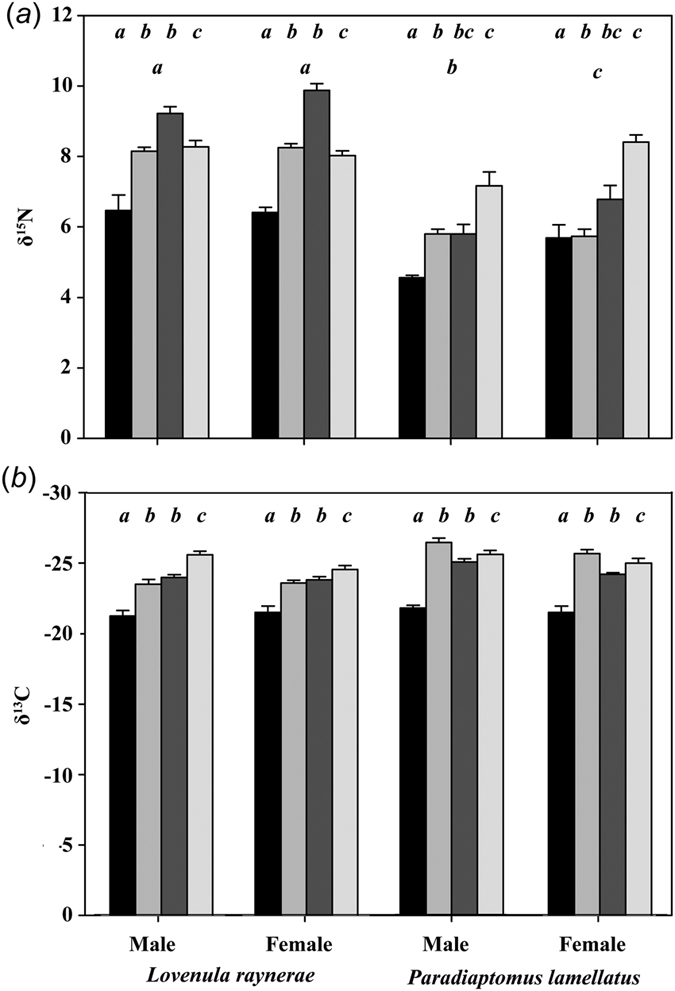
Temporal fluctuations in (**a**) mean δ^15^N (‰) and (**b**) mean δ^13^C (‰) of Paradiaptomidae copepods: *Lovenula raynerae* and *Paradiaptomus lamellatus* over the hydroperiod (surveys 1, 2, 3 and 4) in an ephemeral pond.

**Figure 3:**
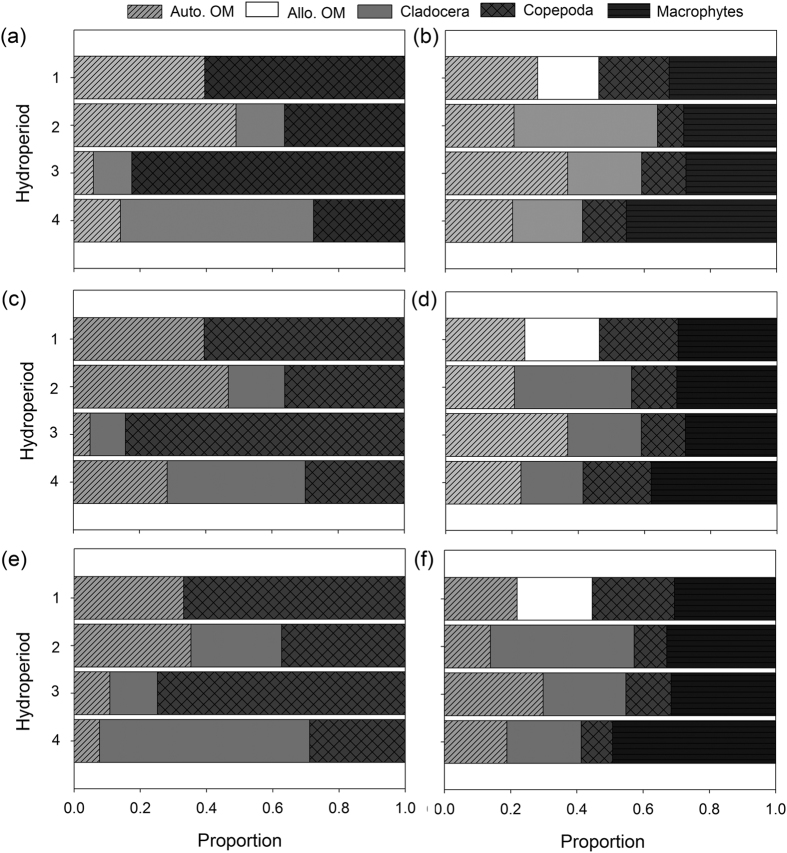
Estimated Stable Isotope Analysis in R (SIAR) mean group possible contributions (%) of food sources to the diets of *Lovenula raynerae*: (**a**) both sexes combined (**c**) female and (**e**) male; and *Paradiaptomus lamellatus*: (**b**) both sexes combined (**d**) female and (**f**) male. Note: Cladocera at survey 1 were grouped together with Copepoda due to similarity and large overlap in isotopic signatures, hence the absence of the former.

In total, 59 *L. raynerae* (30 female, 29 male) and 49 *P. lamellatus* (25 female, 24 male) were analysed for stable isotopes. Significant (*p* < 0.05) variation was observed for *Lovenula raynerae* and *Paradiaptomus lamellatus* species stable isotope values over the course of the hydroperiod (see Figs 3 and S1) and we attribute the stable isotope values and population metrics changes to actual diet alteration. We found significant differences between individual species for both δ^13^C (F = 52.31, *p* < 0.001) and δ^15^N (F = 260.74, *p* < 0.001) and also a significant interaction for species and hydroperiod, for both, δ^13^C (F = 14.82, *p* < 0.001) and δ^15^N (F = 32.26, *p* < 0.001). *Lovenula raynerae* δ^15^N values were consistently significantly higher than those for *P. lamellatus*, whereas δ^13^C values were significantly higher in *P. lamellatus* compared to *L. raynerae* (*p* < 0.001; Fig. 2).

For *L. raynerae* intraspecific variation, no differences were observed for sex and sex × hydroperiod interactions for both the δ^13^C and δ^15^N (Table 1). However, we found significant differences over the hydroperiod for both δ^13^C (F = 41.93, *p* < 0.001) and δ^15^N (F = 75.47, *p* < 0.001). Survey 1 had the depleted δ^15^N and enriched δ^13^C for both sexes (see Fig. 2). A decreasing trend in *L. raynerae* males and females δ^13^C values from survey 1 to 4 was observed, whereas δ^15^N values became enriched from survey 1 to 3 before depleting at survey 4 for both sexes (Fig. 2). With regard to *P. lamellatus*, we observed significant differences (*p* < 0.01) in sex and hydroperiod over the course of the study (Table 1). A significant interaction for sex × hydroperiod for δ^15^N (F = 2.91, *p* = 0.046) was observed, while δ^13^C was shown to be similar (F = 0.22, *p* = 0.885) over time. The *P. lamellatus* δ^15^N became enriched for both sexes over the course of the hydroperiod, with females having more enriched values (see Fig. 2), whereas no clear trend was observed for δ^13^C in both sexes. Similar to *L. raynerae, P. lamellatus*, enriched δ^13^C and depleted δ^15^N values were evident at the start of the study for both sexes (Table 1).

### Copepod mixing models

The mixing models used to identify food sources assimilated by the copepods are based on Bayesian statistics and, the results provide only estimates of important dietary components. As such, any results derived from such models (e.g. SIAR, SIBER) should be interpreted with caution. These models, however, suggested that *Lovenula raynerae* fed mostly on zooplankton, while *P. lamellatus* fed predominantly on autochthonous and allochthonous organic matter (Fig. 3a,b). Copepoda (i.e. *Mesocyclops* spp., *P. lamellatus* and nauplii) were highlighted as a possible portion of the diet for *L. raynerae* female at survey 1 (~60%) and 3 (~85%), with autochthonous organic matter (i.e. POM) and Cladocera (i.e. *Daphnia* spp., *Daphnia longispina, Kurzia* spp.) being the most important contributor at survey 2 (~45%) and 4 (~ 40%; Fig. 3c). For *L. raynerae* male, Copepoda were identified as the most important dietary item during survey 1 (~65%), 2 (~35%) and 3 (~75%), with Cladocera being an important estimated food source at survey 4 (65%). Overall, autochthonous organic matter was also identified as the second most important dietary food resource at survey 1 and 2 for the both *L. raynerae* sexes (Fig. 2e). Autochthonous organic matter was shown to comprise between 5–45% of the diet in females and between 8–35% in males, with zooplankton (Copepoda and Cladocera) also forming an important proportion of the *L. raynerae* diet (Fig. 3a,c and e).

**Figure 4:**
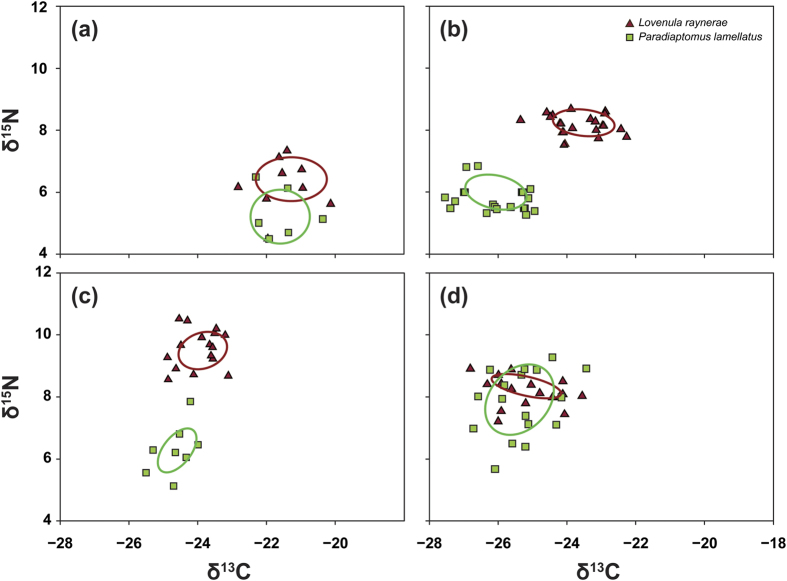
Stable Isotope Bayesian Ellipses in R (SIBER) output for the Paradiaptomidae copepod individual species: (**a**) survey 1, (**b**) survey 2, (**c**) survey 3 and (**d**) survey 4. The ellipse area represents the calculated isotopic feeding niche widths of each species.

Allochthonous organic matter (i.e. detritus and grass – *Sporobolus africanus*) was only available as a potential estimated food resource (~22%) for both *P. lamellatus* sexes during survey 1 (see Figs 3d and S1). In survey 1 and 4, however, aquatic macrophytes were identified as the most important estimated food resource in the diet of female *P. lamellatus*, followed by autochthonous organic matter which contributed about 23% (Table 1). Cladocera were identified as an important estimated food resource in survey 2, contributing 35% for the females. The mixing models suggested that *Paradiaptomus lamellatus* males mainly consumed aquatic macrophytes in survey 1 (~30%), 3 (~30%) and 4 (~50%), with Cladocera forming an important estimated dietary food resource at survey 2 (~40%; Fig. 3f). Overall, zooplankton (i.e. Cladocera, Copepoda – *Mesocyclops* spp. + nauplii) was estimated at contributing between 25–50% of the diet in females and between 25–50% in males, with plants forming an important estimated food resource for *P. lamellatus* (Fig. 4a,d and f).

### Population metrics

The SEAc of *L. raynerae* and *P. lamellatus* species overlapped during low water periods associated with surveys 1 (isotopic niche area overlap 8.8%) when the water had just filled up, and 4 (87.7%), when water was very low. No overlaps were, however, observed during surveys 2 and 3 when water levels were deeper (Fig. 4a,d). The SEAc of *L. raynerae* decreased from survey 1 (1.33) to 2 (0.79) before increasing up to survey 4 (1.30), while *P. lamellatus* gradually decreased from survey 1 (1.48) to 3 (1.04) before significantly increasing at survey 4 (2.71; Fig. 4, Table 2). Inundated terrestrial vegetation would increase with pond depth and could have facilitated food resource diversity for *P. lamellatus*, by providing more access to allochthonous organic matter during periods where the pond was at its deepest. *Lovenula raynerae* had general high dNr and dCr when compared to *P. lamellatus* (Fig. 4, Table 2). The diversity of *L. raynerae* diet, measured as the mean distance to centroid (CD) was general constant before increasing at survey 4. For *P. lamellatus*, the CD was high at survey 1 and 2, due to increased more access to allochthonous organic matter and macrophytes after the pond filled with water (Table 2). The high CD, MNND, SNND and SEAc for *P. lamellatus* suggest that this species was more of an opportunist feeder, while *L. raynerae*, which had lower CD, MNND, SNND and SEAc was more specialised in its feeding.

**Figure 5:**
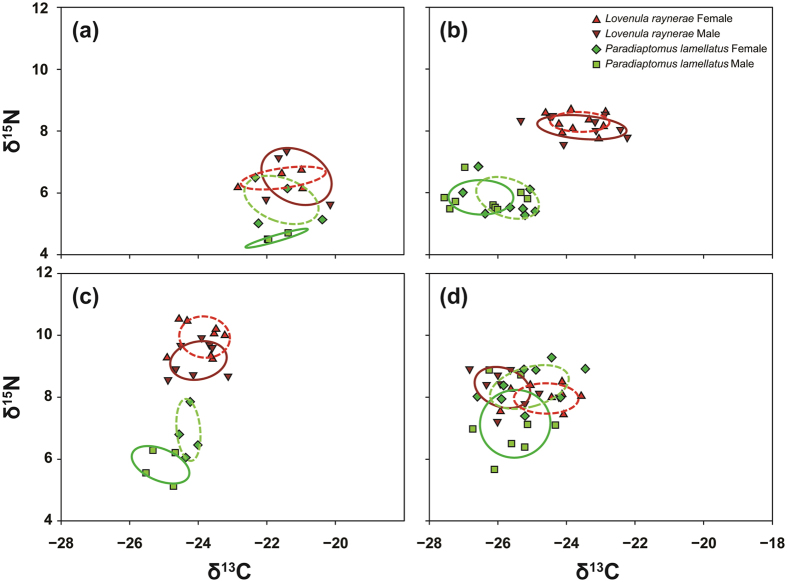
Stable Isotope Bayesian Ellipses in R (SIBER) output for the Paradiaptomidae copepod individual sexes: (**a**) survey 1, (**b**) survey 2, (**c**) survey 3 and (**d**) survey 4. The ellipse area represents the calculated isotopic feeding niche widths of each sex.

The SEAc of *L. raynerae* males and females overlapped throughout the course of the hydroperiod. However, the degree of overlap between the isotopic niche area for both sexes decreased from survey 1 (95%) to survey 4 (35%; Fig. 5; Table 2). The SEAc of *L. raynerae* females general increased from survey 1 to 4, with males exhibiting a decreasing trend over time, with the exception of survey 4 where a slight increase in SEAc was observed (Fig. 5, Table 2). This may have been facilitated by the high zooplankton diversity towards the end of the hydroperiod (Fig. 2). *Lovenula raynerae* males generally had high dNr, dCr, MNND, SNND and CD when compared to females (Table 2), suggesting that the males were more opportunistic or generalist and females more specialist feeders.

*Paradiaptomus lamellatus* males and females SEAc overlapped at survey 2 (70%) and 4 (53%), with zero degree of isotopic niche area overlap for both sexes being observed at surveys 1 and 3 (Fig. 5a–d; Table 2). No clear trends for dNr, dCr, MNND, SNND and CD were observed for both *P. lamellatus* sexes; however, males generally had a high SEAc as compared to females (Fig. 5, Table 2). A low (0.01) and high (2.42) SEAc was observed for the males at surveys 1 and 4, respectively. Contrary to those results of *L. raynerae*, female *P. lamellatus* generally exhibited higher dNr, dCr, MNND, SNND and CD compared to males (Table 2), suggesting that the females were more generalist feeders and males more specialist feeders.

There was a positive correlation between *P. lamellatus* species SEAc and the water depth, which is a proxy of survey time (*r*_4_ = 0.99, *p* = 0.006), which suggests that a high water level could have improved resource diversity by providing access to allochthonous organic matter within the pond margins (i.e. shallow zones). There was significant inverse relationship between water depth and *L. raynerae* individual species TA (*r*_4_ = −0.95, *p* = 0.048), *L. raynerae* female TA (*r*_4_ = −0.99, *p* = 0.015) and SNND (*r*_4_ = −0.96, *p* = 0.041). However, most of the copepod isotopic population metrics were not significantly correlated with hydroperiod.

## Discussion

The present study, using key organisms from a model aquatic ecosystem, showed that the evolution of food web complexity over time is likely to have implications for specialisation at multiple levels, even for pioneer species. Not only was the development of specialisation evident among the two Paradiaptomidae copepod species, but sex-specific specialisation within each species was also shown to occur, particularly for the more omnivorous *Paradiaptomus lamellatus*. Much of the specialisation corresponded to increases and decreases in the overall isotopic niche size of the planktonic foodweb over time, suggesting that resource availability is the main driver of specialisation in these systems.

Our trophic structure results based on stable isotope analysis showed large differences in trophic structure over the course of the hydroperiod, with trophic structure length being shorter in survey 1 (i.e. low trophic complexity) compared to survey 3 (i.e. high trophic complexity) based on TA and SEAc. The suggested differences in energy pathways as highlighted by the survey foodweb shapes, is described by the convex hull shapes and community metrics (i.e. SEAc; see Fig. 1). The findings further suggest that trophic webs are characterised by multi-chain omnivory, with one top predator (i.e. *Lovenula raynerae*) integrating the different carbon sources fuelling the foodweb. However, at survey 4, the occurrence of a combination of multi-chain and single-chain omnivory, and the resultant more complex energy transfer pathways, might explain the community metrics and the shape observed (see Vadeboncoeur *et al*.^25^, Meerhoff *et al*.^26^). In addition, there was an overall carbon variation in the planktonic foodweb over the hydroperiod. This was likely a result of an increase input and uptake of ^13^C depleted organic matter, as a result of the consistent decaying of submerged plant material associated with the filling up of the recently dry vegetated area and allochthonous organic matter input, as has been shown in other studies (see Bouillon *et al*.^27^, Dalu *et al*.^28^, Jha and Masao^29^). For the dCr (i.e. carbon range) and dNr (i.e. nitrogen range), we managed to find differing values across survey trophic webs indicating changes in carbon source and trophic level diversity. The dCr was significantly higher in survey 3, pointing to a mixture of simultaneously occurring strategies where some taxa have a lower integration of carbon sources, while other co-occurring taxa integrate several carbon sources^30^. A high functional redundancy was observed due to closer SNND, meaning that species occupied different trophic web positions over the course of the hydroperiod.

While the dietary niche overlap was largest among the target species at the beginning and end of the hydroperiod, the niche space in which this overlap occurred differed, with the copepods exhibiting carbon depletion and nitrogen enrichment between the first and last sampling event. Interspecific competition between *Lovenula raynerae* and *Paradiaptomus lamellatus* occurred predominantly between the first and second sampling period whereby the former exhibited considerable enrichment in nitrogen and the latter depletion in carbon over time. Between events two and four, however, the isotopic niche spaces of the two species slowly converged, with both nitrogen and carbon levels of *L. raynerae* depleting over time, and *P. lamellatus* levels enriching towards the end of the study. With regard to intra-specific specialisation, the data suggests that females are more predatory than males given that they had higher nitrogen values at certain periods. While the overall isotopic niche of *Lovenula raynerae* decreased in size between surveys 1 and 2, there was little intra-specific specialisation between the sexes of this species during this period. Intra-specific specialisation only seemed to gain momentum between the second and fourth sampling event for *L. raynerae*. For *P. lamellatus*, however, this difference was observed at the first sampling event, with increased homogenisation occurring by the second period. It is not clear what the driver of this particular observation was, but it was likely a result of an increase in small cladoceran availability during this period, given that both sexes seemed to incorporate this available prey during sampling period 2. *Paradiaptomus lamellatus* sex-specific specialisation was, however, evident again by sampling event three. By the last sampling event of the study, not only had *P. lamellatus* male and female isotopic niche space converged, but the niche space of the species also overlapped with that of *L. raynerae*.

The relative contribution based on SIBER analyses of potential food resources to the diet of copepods shifted throughout the hydroperiod, supporting our postulation regarding *L. raynerae* and *P. lamellatus* dietary shifts over time. While these results need to be interpreted with caution given that the outputs are proxies for assimilated foods, the models are still useful in highlighting differences in diets. For *P. lamellatus*, macrophytes and allochthonous material represented the most important assimilated resources throughout the study, with the relative contributions of these two food types varying over time. Interestingly, during the second sampling event, Cladocerans contributed more to the male and female *P. lamellatus* diet, likely as a result of increased availability through hatching of dormant eggs during this time. In contrast, Copepoda (*P. lamellatus* + nauplii) was the most important assimilated resource for the predatory *L. raynerae* relative to the other resources throughout the hydroperiod with the exception of the final sampling event where Cladoceran contributions dominated. While autochthonous material and Cladocerans were also identified as important, the prevalence of copepods in the diet of *L. raynerae* indicates that in addition to this species interacting with *P. lamellatus* through competition, the two species also engage in predator-prey interaction with the former feeding on the latter. This was to be expected as previous work on the species have highlighted that *L. raynerae* is a more efficient predator than *P. lamellatus* and that the former likely feeds on the latter in these environments^18^,^20^. An additional consideration in this study, particularly for the more predatory *L. raynerae* is that of cannibalism. While cannibalism presents a challenge in isotopic studies^20^,^31^,^32^, it is highly likely that *L. raynerae* also forages on conspecifics and that *P. lamellatus* does not. Should the larger female *L. raynerae* incorporate males of the species into their diet, this could result in the homogenisation of isotopic signatures and mask potential differences between the sexes in this regard. Indeed, in the present study, intra-specific specialisation was more evident for the omnivorous *P. lamellatus* than for the predatory *L. raynerae*.

Aside from predator-prey interactions between *L. raynerae* and *P. lamellatus*, it is unclear whether observed trends in trophic signatures where a result of indirect antagonistic interactions between the species. Quantitative population metrics derived for each species using the SIBER and SIAR mixing models suggest that there may be important interaction occurring between the two copepod species. The reduced diversity of *P. lamellatus* diet (CD) increased individual isotopic niche packing (SDNND) and limited the exploited resources total range (dCr), while decreasing the trophic levels numbers utilised (dNr). In contrast, *P. lamellatus* did not affect any isotopic metrics of *L. raynerae*, indicating that *P. lamellatus* presence had little impact on *L. raynerae* trophic ecology. Wasserman *et al*.^18^ postulated that *L. raynerae* may out-compete *P. lamellatus* for food given that the former consumes more and is better at finding prey at low densities. We postulate that inter-specific antagonism may be a consideration driving intra-specific feeding specialisation

Like many populations, the copepod (*L. raynerae* and *P. lamellatus*) individual sexes differed slightly in their diets. Therefore, an assessment of the magnitude and temporal consistency of such intraspecific copepod diet variation is needed to understand its importance. As with all organisms, the copepods experienced differences over the course of the hydroperiod that affected their foraging behaviour and diet resulting in increased magnitude of individual copepod sex variation. These observations were similar to those of Novak and Tinker^33^, who showed strong seasonal cycles in the diet in relation to sea otter sex. There are however few other examples that highlight this process.

Unlike in most aquatic ecosystems where acquiring representative samples of entire food webs is difficult, ephemeral ponds are particularly convenient in this regard. Given their small size, well defined boundaries and occurrence of a discrete wet phase, these systems are ideal for testing aspects relating to aquatic trophic ecology. In the present study, a combination of quantitative population metrics derived from species using the Stable Isotope Bayesian Ellipses in R and Stable Isotope Analysis in R models revealed complex interactions between *L. raynerae* and *P. lamellatus* and their resources within the ephemeral pond, highlighting the importance of demographic and species level considerations in foodweb dynamics. Unfortunately, no data relating to background dissolved inorganic carbon, dissolved inorganic nitrogen and nitrogen concentrations were collected in this study. As such, we have no actual or true measure of the basal resources that were available for primary producers and/or consumers. However, since POM (CN ratio range 9 to 12) and sediment (CN ratio range 12 to 16) ratios observed over the study period did not vary greatly, it is likely that there were no major additions in organic matter into the system, hence the system can be considered to be driven by autochthonous organic matter, as was found in a study by Dalu *et al*.^20^. As such, we propose that the population metrics and stable isotope mixing models employed provide meaningful results for the present study based on the POM isotope values, signifying that the changes in copepod niche size and position were likely a consequence of changes in the proportion and identity of assimilated resources. As such, the present study contributes to the understanding of specialisation in relation to foodweb complexity, not only in ephemeral ponds, but in aquatic ecosystems in general. Thus, future studies should assess the contributions of δ^13^C of dissolved inorganic carbon (δ^13^C–DIC) and δ^15^N of particulate organic carbon (δ^15^N–PN) as this might play an important role as zooplankton community’s food sources.

## Materials and Methods

### Study site

**Figure 6:**
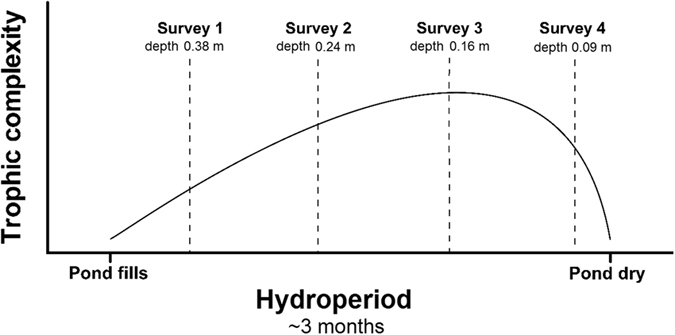
Outline of the isotopic sampling surveys (hashed lines) over the course of the ponds hydroperiod in the present study, in relation to a conceptual model outlining expected evolution of trophic complexity (solid line) over an ephemeral pond’s hydroperiod (modified from O’Neill and Thorp^15^). The present study was conducted in 2015, whereby the pond filled in the middle of June, with surveys 1, 2, 3 and 4 conducted in late June, July, August and September, respectively. The pond dried out in early October 2015 and the water depth for each sampling event is highlighted next to the survey times.

The study was conducted between May and August 2015 over the course of an ephemeral pond’s entire hydroperiod. The pond was situated at Burnt Kraal (33′15°S, 26′26°E) in the south–eastern temperate region of South Africa. The ephemeral pond (temporary pond) measured 40.5 m (length) × 38.5 m (width) × 0.38 m (depth) at maximum depth during the present study. The region has mean summer and winter daily temperatures of 20.3 °C and 12.3 °C, with rainfall (mean annual rainfall ~680 mm) evenly distributed over the entire catchment^34^. Sampling was conducted at four discrete periods over the course of a pond’s hydroperiod. The first sampling event took place on 24 June, two weeks after a large rainfall event that rapidly filled the dry pond (Fig. 6). Sampling event two, three and four were then conducted on 27 July, 29 August and 23 September, respectively as the pond was slowly drying out. For the first survey, the depth of the pond at the deepest point was 0.38 m, reducing to 0.24 m, 0.16 m and 0.09 m for surveys 2, 3 and 4, respectively. Within two weeks of the final sampling event the pond was completely desiccated.

### Sample collection and processing

Stable isotope samples for *Paradiaptomus lamellatus* and *Lovenula raynerae* and their potential prey (Copepoda spp., Cladocera spp., particulate organic matter (POM), detritus, macrophytes, terrestrial grass and dung) were collected on each sampling period at midday. Zooplankton (i.e. *Daphnia* spp., *Daphnia longispina, Daphnia magna Kurzia* spp., *Mesocyclops* spp., nauplii, *L. raynerae* and *P. lamellatus*) were collected by towing a 32 cm diameter, 63 μm mesh size zooplankton net horizontally through the water column. All zooplankton were identified to the lowest taxonomic resolution using keys by Day *et al*.^19^, Suárez-Morales *et al*.^21^, Day *et al*.^35^, Fernando^36^ and placed into toluene cleaned Eppendorf tubes. Both *Lovenula raynerae* and *P. lamellatus* were sexed before being placed into labelled toluene cleaned Eppendorf tubes. For both species, sex was determined using the diagnostic features of the shape of the right antennule and the caudal rami^21^,^35^ (see Figure S2 for more details).

Four surface water samples, 0–20 cm depth, were collected using 20 L containers for the determination of particulate organic matter (POM, < 500 μm size). The POM water was then further filtered through pre–combusted (450 °C, 5 h) Whatman GF/F filters. All visible zooplankton on the filters, were removed with forceps under a dissecting Olympus microscope operated at 100x magnification. Each GF/F filter was then placed in a separate labelled pre–combusted (450 °C, 5 h) aluminium foil bags.

Sediment was collected using a van Veen grab, four independent sediment samples were collected (bite depth ~1–5 cm) and placed into sterile plastic bags for laboratory analysis. Detritus (*n* = 4) and horse dung (*n* = 4) were obtained from the surface sediment within the pond by hand picking and all samples were placed in separate labelled zip-lock bags. In the laboratory, all samples in zip-lock bags were placed into separate, labelled pre–combusted aluminium foil bags. Macrophytes (i.e. *Cyperus* sp., *Crinum compatunalatum, Mersalea* sp., *Laurembergia repens* subsp. *brachypoda*) and terrestrial grass (*Sporobolus africanus*) leaves were collected by hand (*n* = 3–5, each species) and placed in labelled zip-lock bags.

All plant material (detritus, macrophytes, grass, POM), dung, sediment and zooplankton (*n* = 3–16) samples were oven dried at 60 °C for 72 hrs before being further ground to a fine homogeneous powder using a mortar and pestle. To obtain sufficient amounts of material to conduct the stable isotope analysis, zooplankton (excluding *Daphnia magna, L. raynerae* and *P. lamellatus*) were pooled using individuals of a similar group, Cladocera (*Daphnia* spp., *D. longispina* and *Kurzia* spp.) and Copepoda (*Mesocyclops* spp. and nauplii). Aliquots of approximately 0.6–0.7 mg (animals) and 1–1.2 mg (plant material, dung, sediment) were weighed into tin capsules that were pre–cleaned in toluene.

### Stable isotope sample analysis

Stable isotope analysis was carried out using a Flash EA 1112 Series coupled to a Delta V Plus stable light isotope ratio mass spectrometer via a ConFlo IV system (Thermo Fischer, Bremen, Germany), housed at the Stable Isotope Laboratory, University of Pretoria. Merck Gel (δ^13^C = −20.57‰, δ^15^N = 6.8‰, C% = 43.83, N% = 14.64) standards and blank sample were run after every 12 unknown samples. All results were referenced to Vienna Pee–Dee Belemnite and to air for carbon and nitrogen isotope values, respectively. Results were expressed in delta notation using a per mille scale from the standard equation 1:


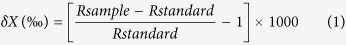


where X = ^15^N or ^13^C and R represents ^15^N/^14^N or ^13^C/^12^C, respectively. Average analytical precision was <0.15‰ for δ^13^C and <0.1‰ for δ^15^N.

The trophic positions of the zooplankton in the pond were estimated using the formula of Vander Zanden *et al*.^37^ (equation 2):


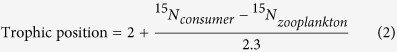


where δ^15^N_consumer_ is the measured consumer δ^15^N for which TP needs to be estimated and δ^15^N_zooplankton_ is the average δ^15^N of the primary consumer (mean of Cladocerans) at a particular hydroperiod and 2.3 is the trophic fractionation for δ^15^N. The level 2 was consequently attributed, empirically, to zooplankton i.e. Cladocera^20^.

### Data analysis

The data was, prior to analyses, tested for normality and homeogeneity of variance using Q-Q plots and Levene’s tests, respectively^38^,^39^. As data did not violate parametric assumptions, analysis of variance (ANOVA) was used in R^40^, assessing the differences in isotopic signatures (δ^15^N, δ^13^C) between the two species and between sexes within each species over time. Tukey Post Hoc tests were run to test the difference amongst the levels within the factors used (species, sex, hydroperiod).

To investigate trophic structure of *L. raynerae* and *P. lamellatus* found in the ephemeral pond, quantitative population metrics^41^ were derived for the each species using the Stable Isotope Bayesian Ellipses in R (SIBER; Jackson *et al*.^42^) model. Layman metrics included dNr (δ^15^N range) which provide information on the community trophic length and dCr (δ^13^C range), providing a univariate measure of estimate of the diversity of basal resources; mean distance to the centroid (CD), which provides a description of trophic diversity (niche width and species spacing); mean nearest neighbour distance (MNND), providing a measure of density and clustering of species within the community; standard deviation of nearest neighbour distance (SDNND), which provides a measure of trophic evenness and packing; and standard ellipse area (SEAc), which provides a bivariate measure of mean core isotopic niche width (see Layman^41^, Jackson *et al*.^42^ for detailed methodology). To allow comparisons between species populations with varying sample sizes, all metrics were bootstrapped (n = 9999) and a small sample size correction for improving accuracy to SEA values is indicated by the subscript ‘c’^42^.

Estimates of the relative contribution of dietary resources assimilated by *L. raynerae* and *P. lamellatus* (individual sexes) in the ephemeral pond were obtained using bivariate, single–group mixing models in the Stable Isotope Analysis in R model (SIAR; Parnell *et al*.^43^). All collected food sources with similar isotopic values were grouped together to ensure all potential food sources that were isotopically distinct and each zooplankton species was assigned a list of estimated potential food sources (see Table 3). Fractionation factors of δ^15^N 2.3 ± 0.18 and δ^13^C 0.5 ± 0.13 were used for all animals, and δ^15^N 1.1 ± 0.29 and δ^13^C –0.21 ± 0.21 for all acidified samples, respectively (see Dalu *et al*.^20^, McCutchan *et al*.^44^). The models used food source data for each of the different hydroperiods (as these changed over time).

## Additional Information

**How to cite this article:** Dalu, T. *et al*. Sex and species specific isotopic niche specialisation increases with trophic complexity: evidence from an ephemeral pond ecosystem. *Sci. Rep.* **7**, 43229; doi: 10.1038/srep43229 (2017).

**Publisher's note:** Springer Nature remains neutral with regard to jurisdictional claims in published maps and institutional affiliations.

## Supplementary Material

Supplementary Files

## Figures and Tables

**Table 1:** Analysis of variance (ANOVA) results for *Lovenula raynerae* and *Paradiaptomus lamellatus* showing significant differences between the two species, sex within each species, hydroperiod and interactions between species and hydroperiod as well as sex and hydroperiod.

	δ^15^N			δ^13^C		
	DF	F	*p*	DF	F	*p*
**Interspecific variation**						
Species	1	**260.74**	**<0.001**	1	**52.31**	**<0.001**
Species:Hydroperiod	3	**32.26**	**<0.001**	3	**14.82**	**<0.001**
**Intraspecific variation**						
***Lovenula raynerae***						
Sex	1	1.18	0.282	1	2.55	0.116
Hydroperiod	3	**75.47**	**<0.001**	**3**	**41.93**	**<0.001**
Sex:Hydroperiod	3	2.56	0.066	3	2.03	0.121
***Paradiaptomus lamellatus***						
Sex	1	**14.84**	**<0.001**	**1**	**10.35**	**0.003**
Hydroperiod	3	**35.29**	**<0.001**	**3**	**52.78**	**<0.001**
Sex:Hydroperiod	3	**2.91**	**0.046**	3	0.22	0.885

DF – degrees of freedom.

**Table 2:** Estimated Layman’s metrics describing populations of the Paradiaptomidae copepod individual species (combined sexes) and different sexes over the course of a hydroperiod (surveys 1–4).

Survey	Sex	N	dNr	dCr	TA	CD	MNND	SNND	SEAc
***Lovenula raynerae***									
1	Combined sexes	8	1.5 (1.5–1.7)	2.1 (1.9–2.7)	1.5 (1.1–2.0)	0.8 (0.7–0.9)	0.29 (0.20–0.38)	0.40 (0.34–0.48)	1.4 (0.9–1.8)
2	Combined sexes	17	1.0 (0.9–1.1)	2.7 (2.4–3.1)	1.7 (1.4–2.0)	0.8 (0.7–0.8)	0.13 (0.1–0.16)	0.2 (0.17–0.24)	0.8 (0.7–0.9)
3	Combined sexes	18	1.8 (1.8–2.0)	1.7 (1.7–1.8)	2.09 (1.8–2.5)	0.7 (0.7–0.8)	0.15 (0.12–0.19)	0.24 (0.2–0.27)	1.1 (0.9–1.2)
4	Combined sexes	16	1.6 (1.5–1.7)	2.9 (2.7–3.3)	2.7 (2.3–3.1)	0.9 (0.8–1.0)	0.17 (0.13–0.21)	0.25 (0.22–0.28)	1.3 (1.1–1.5)
1	Female	4	0.6 (0.6–0.6)	1.8 (1.6–1.9)	0.6 (0.6–0.6)	0.7 (0.6–0.8)	0.04 (0.004–0.05)	0.11 (0.01–0.17)	0.6 (0.5–0.7)
2	Female	9	0.8 (0.8–0.9)	1.6 (1.4–1.8)	0.8 (0.6–0.9)	0.6 (0.5–0.6)	0.19 (0.14–0.24)	024 (0.21–0.28)	0.6 (0.5–0.7)
3	Female	9	1.2 (1.2–1.3)	1.4 (1.3–1.7)	1.0 (0.6–1.4)	0.7 (0.6–0.8)	0.19 (0.11–0.28)	0.33 (0.15–0.46)	0.9 (0.7–1.2)
4	Female	4	0.9 (0.9–1.1)	2 (1.8–2.4)	1.1 (0.8–1.3)	0.7 (0.6–0.8)	0.25 (0.17–0.32)	0.33 (0.27–0.67)	0.9 (0.7–1.1)
1	Male	4	1.7 (1.7–1.7)	1.8 (1.9–1.9)	1.5 (1.6–1.6)	1.0 (0.9–1.1)	0.05 (0.004–0.12)	0.14 (0.01–0.39)	1.6 (1.4–1.9)
2	Male	8	0.8 (0.8–1.0)	2.7 (2.3–3.1)	1.2 (0.8–1.6)	0.9 (0.8–1.0)	0.21 (0.14–0.28)	0.33 (0.29–0.4)	0.9 (0.7–1.2)
3	Male	9	1.2 (1.1–1.4)	1.5 (1.3–1.8)	1.0 (0.7–1.2)	0.7 (0.6–0.7)	0.22 (0.15–0.46)	0.32 (0.25–0.39)	0.9 (0.6–1.1)
4	Male	8	1.4 (1.1–1.7)	2.2 (1.8–2.6)	1.5 (1.0–1.9)	0.8 (0.7–0.9)	0.24 (0.17–0.31)	0.34 (0.27–0.41)	1.1 (0.8–1.5)
***Paradiaptomus lamellatus***									
1	Combined sexes	7	1.8 (1.6–2.0)	1.6 (1.0–2.0)	1.4 (1.03–1.9)	0.8 (0.7–0.9)	0.30 (0.17–0.41)	0.45 (0.38–0.56)	1.5 (1.1–2.0)
2	Combined sexes	17	1.5 (1.5–1.6)	2.5 (2.4–2.7)	2.3 (2.1–2.6)	0.9 (0.8–0.9)	0.13 (0.1–0.16)	0.20 (0.16–0.24)	1.2 (1.0–1.4)
3	Combined sexes	8	2.1 (1.7–2.7)	1.3 (1.2–1.5)	1.2 (0.9–1.5)	0.7 (0.6–0.8)	0.29 (0.19–0.39)	0.39 (0.33–0.45)	1.0 (0.8–1.3)
4	Combined sexes	17	1.5 (2.9–3.6)	2.9 (2.6–3.3)	5.4 (4.8–6.1)	1.2 (1.1–1.3)	0.26 (0.20–0.32)	0.38 (0.34–0.43)	2.7 (2.4–3.1)
1	Female	4	1.5 (1.5–1.5)	1.9 (2.0–2.0)	1.6 (1.7–1.7)	0.9 (0.8–1.0)	0.6 (0.004–0.12)	0.17 (0.01–0.39)	1.5 (1.3–1.7)
2	Female	9	1.3 (0.8–1.6)	1.9 (1.7–2.1)	1.2 (0.9–1.6)	0.8 (0.7–0.9)	0.25 (0.16–0.33)	0.36 (0.3–0.43)	1.0 (0.8–1.3)
3	Female	4	1.8 (1.8–1.8)	0.5 (0.5–0.5)	0.5 (0.5–0.5)	0.6 (0.5–0.7)	0.03 (0.005–0.05)	0.1 (0.01–0.18)	0.4 (0.4–0.5)
4	Female	8	1.6 (1.3–1.9)	2.6 (2.4–3.1)	2 (1.6–2.4)	0.9 (0.9–1.0)	0.3 (0.21–0.39)	0.41 (0.36–0.49)	1.6 (1.3–1.8)
1	Male	3	0.2 (0.2–0.2)	0.6 (0.6–0.6)	0.01 (0.01–0.02)	0.2 (0.2–0.3)	0.01 (0.004–0.01)	0.03 (0.01–0.01)	0.01 (0.01–0.01)
2	Male	8	1.1 (0.6–1.4)	2.2 (2.1–2.4)	1.3 (0.7–1.7)	0.8 (0.7–0.9)	0.18 (0.11–0.23)	0.3 (0.15–0.41)	1.0 (0.6–1.4)
3	Male	4	1.2 (1.2–1.2)	0.8 (0.9–0.9)	0.7 (0.7–0.7)	0.6 (0.5–0.6)	0.03 (0.004–0.06)	0.1 (0.01–0.21)	0.6 (0.5–0.6)
4	Male	9	2.7 (2.5–3.2)	2.0 (1.6–2.4)	2.9 (2.2–3.6)	1.1 (1.0–1.2)	0.39 (0.26–0.51)	0.53 (0.44–0.64)	2.4 (2.0–3.0)

Abbreviations: dNr – δ^15^N range, dCr – δ^13^C range, CD – mean distance to centroid, SDNND – SD mean nearest neighbour distance and SEAc – corrected standard ellipse area. Numbers in parentheses represent the 2.5–97.5% quantile range.

**Table 3:** Assigned potential food sources to the different species of copepods, for analysis in SIAR.

Survey	Copepoda species	
	*Lovenula raynerae*	*Paradiaptomus lamellatus*
1	(**POM + algae**), *P. lamellatus*, **Copepoda (*****Mesocyslops*****sp. + nauplii)**	**(POM + sediment + algae), (detritus + grass)**, Copepoda, *Mersalea* sp.
2	**Cladocera (1) (*****Daphnia*** **spp. + *****Kurzia*** **spp. + *****Daphnia longispina***), *Daphnia magna, P. lamellatus*, POM, Copepoda	*Mersalea* sp., Cladocera (1), *Daphnia magna*, POM, Copepoda, (***C. compatunalatum***** + *****Persicaria*** **sp.)**
3	**Cladocera (2) (*****D. magna**, **Daphnia*** **spp. + *****Kurzia*****spp. + *****Daphnia longispina***), *P. lamellatus*, POM, Copepoda	*Mersalea* sp., Cladocera (2), sediment, POM, Copepoda, (***C. compatunalatum***** + *****Persicaria*** **sp. + *****Cyperus marginatus***)
4	Cladocera (2), *P. lamellatus*, POM, Copepoda	Cladocera (2), sediment, POM, *Laurembergia repens* subsp. *brachypoda*, Copepoda, (***C. compatunalatum + C. marginatus***)

In brackets and bold indicate plants or organisms that were grouped together due to similar isotopic values.
