# Supplementary material for: Sex and species specific isotopic niche specialisation increases with trophic complexity: evidence from an ephemeral pond ecosystem
Source: Sci Rep. 2017 Feb 24;7:43229. doi: 10.1038/srep43229 (PMC5324113; doi:10.1038/srep43229)
Supplement: Supplementary Files [file srep43229-s1.doc]

**Sex and species specific isotopic niche specialisation increases with trophic complexity: evidence from an ephemeral pond ecosystem**

Tatenda Dalu^1, A*^, Ryan J Wasserman^2, A^, Tim J.F. Vink^3^, Olaf LF Weyl^2^

*^1^Zoology and Entomology, Rhodes University, Grahamstown, Eastern Cape, South Africa*

*^2^South African Institute for Aquatic Biodiversity, Grahamstown, Eastern Cape, South Africa*

*^3^Department of Botany, Coastal and Marine Research Unit, Nelson Mandela Metropolitan University, P O Box 77000, Port Elizabeth 6031, South Africa.*

*** ***Corresponding author e–mail:***[*dalutatenda@yahoo.co.uk*](mailto:dalutatenda@yahoo.co.uk)

^A^ These authors contributed equally to this work

# Supplementary files

**
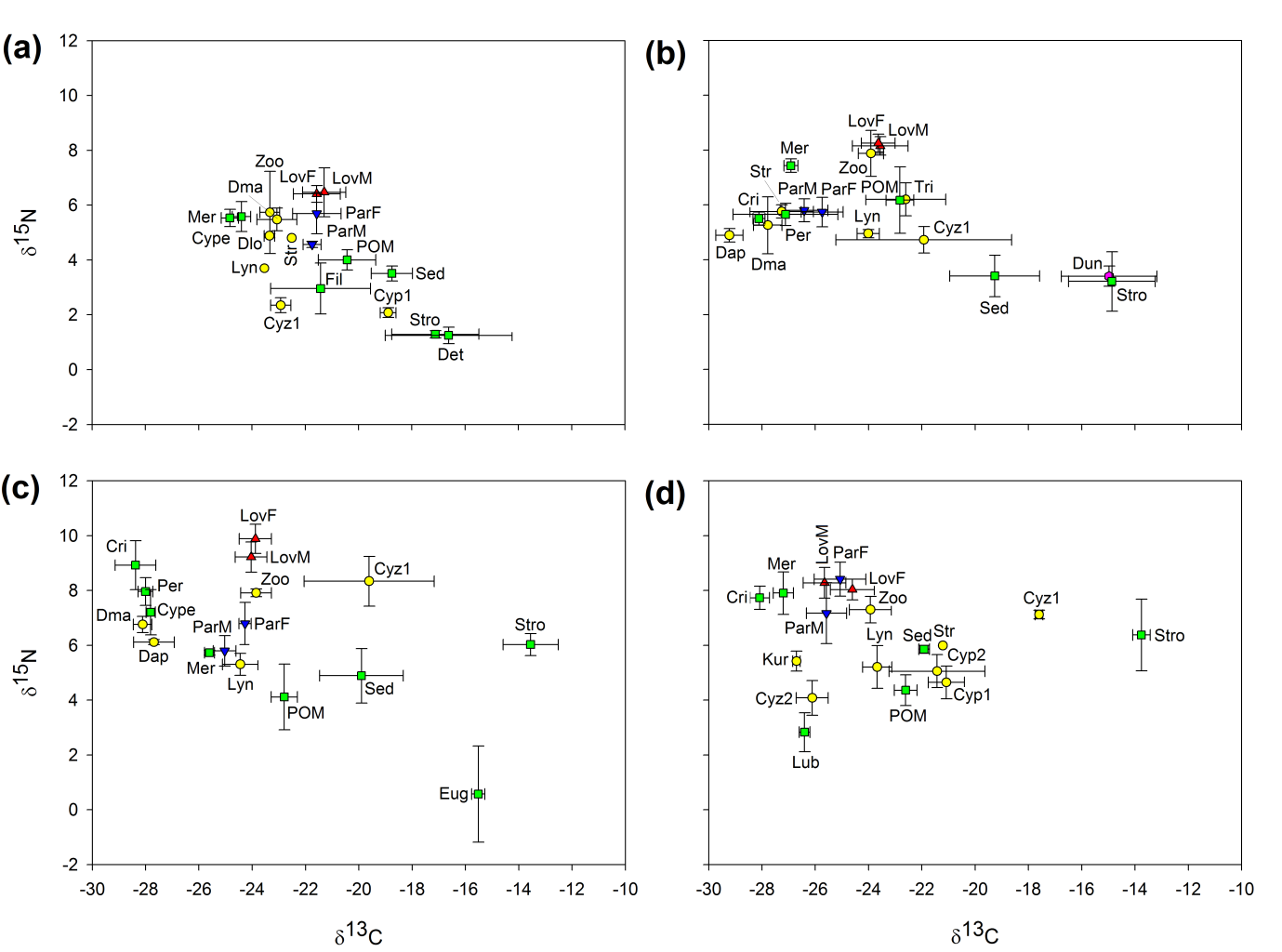
Figure S1.** Stable isotope bi-plots showing variation in trophic structure of potential food resources and copepod species over the course of a hydroperiod: (***a***) survey 1, (***b***) survey 2, (***c***) survey 3 and (***d***) survey 4. The error bars represent the standard deviation, Copepoda red triangles: *Lovenula raynerae* male and female – LovM and LovF, Copepoda inverted blue triangles: *Paradiaptomus lamellatus* male and female – ParM and ParF, invertebrates yellow circles: *Cypricercus* sp. 1 *–* Cyp1, *Cypricercus* sp. 2 *–* Cyp2, *Cyzicus* sp. 1 *–* Cyz1, *Cyzicus* sp. 2 *–* Cyz2, *Daphnia longispina –* Dlo, *Daphnia magna –* Dma, *Kurzia* spp. *–* Kur, *Daphnia* (*Kurzia* spp., *D. longispina*) *–* Dap, *Lynceus* sp. 1 *–* Lyn, *Streptocephalus* sp. *–* Str, *Triops granaries* – Tri, *Copepoda* (*Mesocyclops* spp., *naupli*) – Zoo, plants green squares: *Cyperus* spp. – Cype, *Euglenophyta* – Eug, *Filamentous algae –* Fil, *Particulate organic matter –* POM, *Laurembergia repens* subsp.*brachypoda* – Lub, *Marsilea* spp. *–* Mer, *Persicaria* sp. *–* Per, *Sporobolus africanus –* Stro, *Crinum* sp. *–* Cri, sediment *–* Sed, pink circles: horse dung *–* Dun.

**
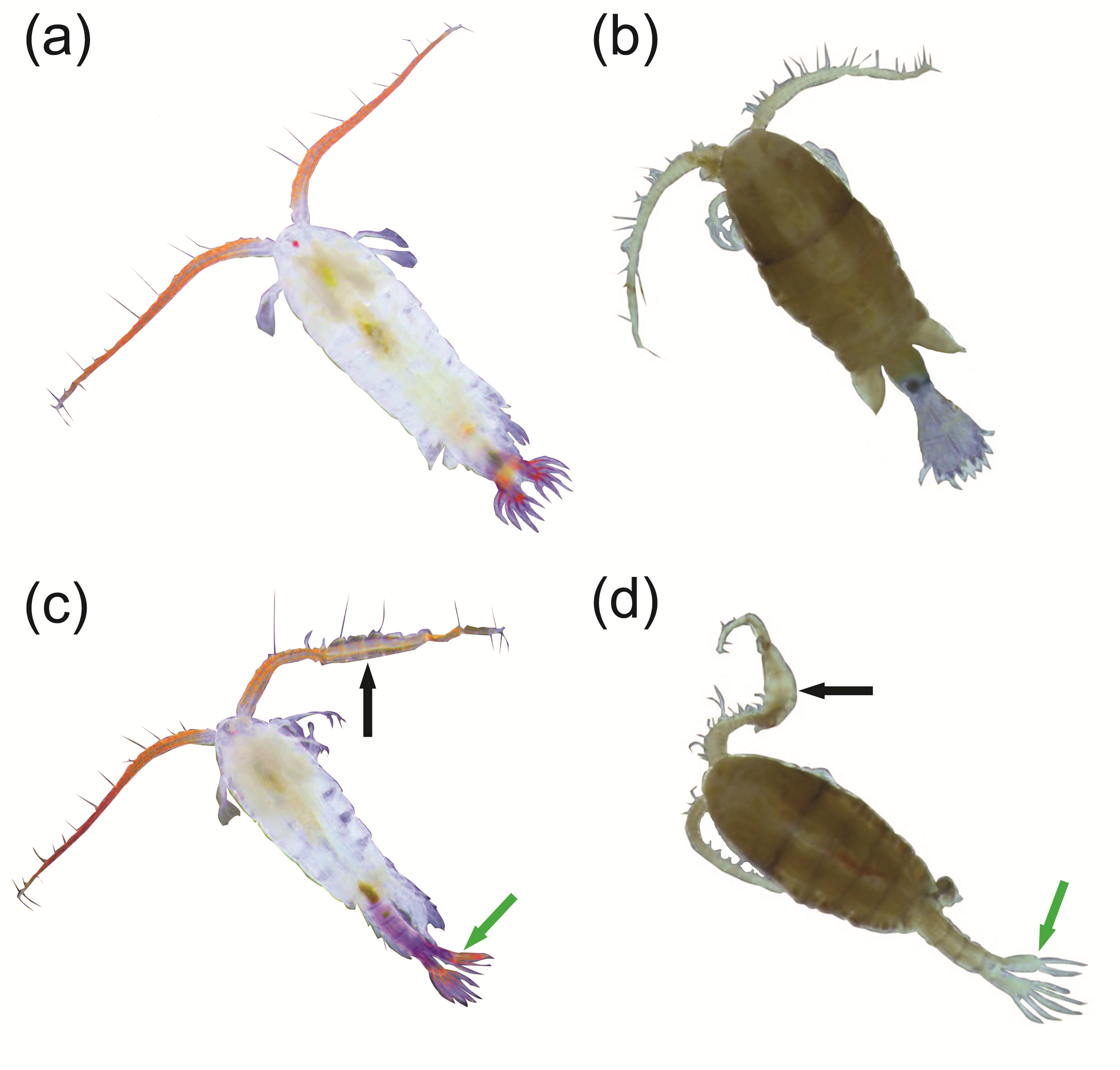
**

**Fig S2.** Doral view of *Lovenula ryanerae* (a, c) and *Paradiaptomus lamellatus* (b, d), highlighting the morphological differencesbetween the female (a, b) and male (c, d) of each. Note the differences in shape of the right antennules (black arrows) and of the caudal rami (green arrows) between the sexes for both copepod species.

**Table S1. Layman’s metrics describing the plankton trophic complexity over the course of a hydroperiod (surveys 1–4). Abbreviations: dNr – δ^15^N range, dCr – δ^13^C range, CD – mean distance to centroid, SDNND – SD mean nearest neighbour distance and SEAc – corrected standard ellipse area. Numbers in parentheses represent the 2.5–97.5% quantile range**

| **Survey** | **dNr** | **dCr** | **TA** | **CD** | **MNND** | **SNND** | **SEAc** |
| --- | --- | --- | --- | --- | --- | --- | --- |
| 1 | 5.7 (5.5–6.0) | 5.2 (5.1–5.3) | 18.5 (17.7–19.6) | 2.0 (1.8–2.1) | 0.19 (0.16–0.22) | 0.31 (0.27–0.36) | 7.6 (6.8–8.3) |
| 2 | 4.7 (4.7–4.9) | 11.0 (10.4–11.9) | 31.6 (29.6–33.9) | 2.5 (2.4–2.6) | 0.17 (0.15–0.20) | 0.33 (0.29–0.37) | 10.2 (9.3–11.1) |
| 3 | 5.7 (5.4–6.0) | 11.3 (10.2–12.4) | 35.2 (33.6–38.4) | 2.5 (2.4–2.6) | 0.23 (0.19–0.27) | 0.47 (0.36–0.57) | 11.6 (10.4–12.9) |
| 4 | 5.7 (5.5–6.0) | 9.0 (9.2–9.4) | 32.9 (32.3–34.7) | 2.2 (2.1–2.4) | 0.20 (0.17–0.23) | 0.37 (0.31–0.42) | 10.2 (9.1–11.3) |
